# Cytochrome P450 2A6 is associated with macrophage polarization and is a potential biomarker for hepatocellular carcinoma

**DOI:** 10.1002/2211-5463.13089

**Published:** 2021-02-05

**Authors:** Tao Jiang, Ai‐song Zhu, Chu‐qi Yang, Chu‐yun Xu, Dan‐qian Yang, Zhao‐huan Lou, Guang‐ji Zhang

**Affiliations:** ^1^ School of Basic Medical Sciences Zhejiang Chinese Medical University Hangzhou China; ^2^ College of Pharmaceutical Science Zhejiang Chinese Medical University Hangzhou China

**Keywords:** arachidonic acid, cytochrome P450, hepatocellular carcinoma, macrophage polarization, tanshinone IIA

## Abstract

Cytochrome P450 2A6 (CYP2A6) is an important metabolic enzyme and is involved in the progression of hepatocellular carcinoma (HCC). However, its specific function and the mechanism of modulation remain to be elucidated. In this study, we found that CYP2A6 is dramatically downregulated in HCC. CYP2A6 expression is closely associated with pathological grading, histologic grade, hepatitis, vascular metastasis, liver inflammation, and worse prognosis. Reduced expression of CYP2A6 contributes to alternative activation of macrophage polarization and impairs macrophage maturation and phagocytosis. Mechanistically, CYP2A6 participates in arachidonic acid metabolism, initiates 20‐hydroxyeicosatetraenoic acid (HETE) generation, and inhibits epoxyeicosatrienoic acid (EET) generation. Disruption of the equilibrium between 20‐HETE and EETs can induce macrophage polarization, thereby modulating antitumor immunity.

## Graphical abstract

Cytochrome P450 2A6 (CYP2A6) has an antitumor role and is dramatically downregulated in HCC. CYP2A6 participates in arachidonic acid metabolism, initiates 20‐hydroxyeicosatetraenoic acid (HETE) generation, and inhibits epoxyeicosatrienoic acid (EET) generation. Disruption of the equilibrium between 20‐HETE and EETs can induce macrophage polarization, thereby modulating antitumor immunity.

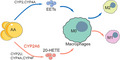

**Abbreviations**
AA: arachidonic acid
BMI: body mass index
CYP2A6: cytochrome P450 2A6
CYP450: cytochromes P450
DFS: disease‐free survival
DMEM: Dulbecco’s modified Eagle’s medium
EET: epoxyeicosatrienoic acid
HCC: hepatocellular carcinoma
HETE: hydroxyeicosatetraenoic acid
HPA: Human Protein Atlas
IHC: immunohistochemistry
IL‐1β: interleukin 1 beta
iNOS: inducible nitric oxide synthase
KEGG: Kyoto Encyclopedia of Genes and Genomes
Met: methoxaline
OB: oral utilization
OS: overall survival
PFS: progression‐free survival
PPARs: peroxisome proliferator‐activated receptors
RFS: relapse‐free survival
TAMs: tumor‐associated macrophages
TCGA: The Cancer Genome Atlas
TCMSP: Traditional Chinese Medicine Systems Pharmacology Database and Analysis Platform
TNF‐α: tumor necrosis factor alpha
TSIIA: tanshinone IIA

Hepatocellular carcinoma (HCC) is a major health challenge for modern society and imposes a heavy burden on healthcare worldwide [1]. The lack of early diagnosis means that the majority of patients with HCC suffer from poor prognoses. Current treatment methods are unsatisfactory, so there is an urgent requirement to identify new targets and corresponding therapeutic drugs.

Immunity and metabolism are two key factors affecting the progression of HCC, and a large number of studies have shown a close relationship between the two [2, 3]. The cytochromes P450 (CYP450) family is a concern in the regulation of metabolism and immunity. CYP450 is a bulky gene superfamily with more than 500 distinctive subtypes identified so far [4]. CYP450 family members can affect tumor progression by metabolizing exogenous and endogenous substances. Cytochrome P450 2A6 (CYP2A6) is an important member of the CYP450 family. However, most studies on CYP2A6 so far have focused on nicotine metabolism [5]. Several studies hint at a previously unappreciated aspect of CYP2A6 in tumor [6, 7, 8], but current understanding of CYP2A6 function in tumor is controversial. Therefore, it is crucial to explore the expression of CYP2A6 in HCC, analyze the possible involvement of CYP2A6 in immune changes, and search for novel potential therapeutic agents. Our previous results showed that multiple extracts of *Salvia miltiorrhiza* could suppress tumor progression [9, 10]. Tanshinone IIA (TSIIA), which is extracted from *S. miltiorrhiza*, is a drug that regulates metabolism and is closely related to the CYP450 family [11, 12]. However, it is not clear if TSIIA regulates CYP2A6 and the possible mechanisms remain to be elucidated.

Combining experimental and bioinformatics approaches, we evaluated the expression of CYP2A6 in HCC and assessed the correlation between the clinical features of HCC and the expression of CYP2A6 to evaluate its potential as a new HCC biomarker. We also linked expression of CYP2A6 to immune cells in an attempt to find evidence of an association between metabolism and immunity.

## Materials and methods

### Expression of CYP2A6 in HCC

The expression of CYP2A6 was investigated using data from multiple databases, including Oncomine (https://www.oncomine.org/), The Cancer Genome Atlas (TCGA) (https://www.tcga.org/), International Cancer Genome Consortium (https://www.icgc.org/), and Gene Expression Omnibus (GEO; https://www.ncbi.nlm.nih.gov/geo/). The mRNA sequencing data were acquired from the aforementioned databases, and the analysis was conducted using the *limma* software package for *R* [13]. The Human Protein Atlas (HPA, https://www.proteinatlas.org/) was used to probe the immunohistochemistry (IHC) of CYP2A6 in HCC tissues and normal tissue.

### Analysis of clinicopathological parameters, immunological status

RNA‐Seq data and related clinical data were collected from the TCGA portal. Data were then grouped according to different clinicopathological parameters, and the expression of CYP2A6 was evaluated. Survival analysis was investigated with a Kaplan–Meier (KM) plot (KM plotter, https://www.kmplot.com/) [14]. To evaluate the relationship between CYP2A6 expression and immune cell abundance, we performed a deconvolution of 22 immune cell types using the CIBERSORT algorithm [15]. The LM22 signature was used as a reference and absolute tumor purity scores were used to assess the abundance of 22 immune cell types in each sample. In addition, the TIMER database (https://cistrome.shinyapps.io/timer/) was also used to identify the correlation between immune cells and CYP2A6.

### Co‐expressed genes with CYP2A6 and related KEGG pathway analysis

Oncomine (https://www.oncomine.org/) was used to obtain CYP2A6 co‐expressed genes and top 300 genes were selected ultimately. Then, STRING V11.0 (https://www.string‐db.org/) was used for Kyoto Encyclopedia of Genes and Genomes (KEGG) pathway analysis of 300 CYP2A6‐related genes.

### Molecular docking of CYP2A6 and TSIIA

Compounds in *S. miltiorrhiza* were retained only if oral utilization ≥ 30 and drug likeness ≥ 0.18 to satisfy criteria suggested by the Traditional Chinese Medicine Systems Pharmacology Database and Analysis Platform (TCMSP) database (https://tcmspw.com/tcmsp.php). By screening the targets of eligible compounds, we can get the potential compounds targeting with CYP450. The crystal structure of compounds was obtained from PubChem database (https://pubchem.ncbi.nlm.nih.gov/). The protein structure of CYP2A6 can be downloaded on Protein Data Bank (PDB, https://www.rcsb.org/), high‐resolution model was selected. Then, the docking was performed by CB‐Dock online tool (http://clab.labshare.cn/cb‐dock/php/).

### Construction and grouping of mouse HCC models

The Hep1‐6 cells (Sibcb, Shanghai, China) were cultured in Dulbecco's modified Eagle's medium (DMEM) supplemented with 10% (v/v) FBS and 1.5 g·L^−1^ NaHCO_3_ in the presence of 5% CO_2_ at 37 °C in humidified chambers. Hep1‐6 cells in the logarithmic phase of growth were digested with trypsin into cell suspensions, and cell concentrations were adjusted to 1 × 10^7^ cells/mL. Four‐week‐old C57/BL mice (*n* = 12) were provided by the Animal Supply Center of Zhejiang Academy of Medical Science [Hangzhou, China; certificate no. SCXK (Zhe) 2014‐0001]. Animal procedures were approved by the Medical Ethics Committee of Zhejiang Chinese Medical University. A transplanted tumor model was established by inoculating 0.2 mL cell suspension under the subcutaneous tissue of the right axilla. After 7 days, mice were divided into control and TSIIA groups. The TSIIA group mice were administered with TSIIA (30 mg·kg^−1^) for 3 weeks. The control group was administered with the same amount of saline. Tumor growth was measured and calculated as *V* = *a *× *b* ^2^/2 (*a*, length; *b*, short diameter) every 3 days. After 3 weeks, mice were anesthetized and sacrificed. Blood was collected, and arachidonic acid (AA) metabolites were detected. Tumor tissue samples and liver samples were harvested for subsequent experiments.

### Culture and grouping of cells

The Hep1‐6 cells were seeded in 24‐well plates. Then, cells were divided into three groups, namely control, TSIIA, and methoxaline (Met). The TSIIA groups were given 10 µm TSIIA; the Met groups were given 10 µm TSIIA and 5 µm CYP2A6 inhibitor Met. After 48‐h culturing, the supernatant was collected. Part of the supernatant fraction was used for the detection of AA metabolites and the other was added to Raw264.7 (Sibcb) as conditioned medium to observe the effects on macrophages. After 48 h of coculture, macrophages were collected for subsequent detection.

### Macrophage polarization analysis

Macrophages in tumors were labeled and identified by flow cytometry. Briefly, fresh tumor tissue was cut into small pieces on ice before digesting with 1 mg·mL^−1^ collagenase I (Biorigin, Beijing, China) and 200 g·mL^−1^ DNase I (Absin, Shanghai, China) at 37 °C for 30 min. Following digestion, the tumor tissue suspension was filtered through a 70‐μm cell filter to harvest the tumor cell suspension, which was then centrifuged at 850 ***g*** for 5 min. Cells were resuspended before incubating with Fc Block (BD, Franklin Lakes, NJ, USA) for 10 min and then stained with conjugated antibodies for 15 min at 4 °C. Macrophages were labeled with PE‐Cy7 anti‐mouse CD45 antibody, AF‐700 anti‐mouse CD11b antibody, BV‐605 anti‐mouse F4/80 antibody (BD Pharmingen). M1 macrophages were labeled with BV‐421 anti‐mouse CD86 antibody (BD Pharmingen), and M2 macrophages were labeled with FITC anti‐mouse CD206 antibody (eBioscience, Waltham, MA, USA).

### Analysis of macrophage phagocytic

The phagocytic ability of macrophage was measured by neutral red uptake. After 48‐h incubating, the conditioned medium was replaced with 200 µL neutral red solutions (dissolved in DMEM at the concentration of 0.05%). After incubation for 2 h, the supernatant was discarded and the cells were washed twice with PBS. Then, cells were lysed by 200 µL cell lysate solution (100 µL EtOH and 100 µL 0.01% acetic acid) at 4 °C for 12 h. The optical density was measured at 490 nm by a microplate reader.

### Evaluation of macrophage viability

CCK8 was used to detect the relative activity of the cells. Macrophages were inoculated in 96‐well plate and were cocultured with conditioned medium for 48 h, and 10 µL CCK8 solution (Boster, Wuhan, China) was added into each well. After incubation in incubator for 1 h, the absorbance was read at 450 nm.

### Analysis of AA metabolites and macrophage‐secreted factor

Arachidonic acid metabolites of 8,9‐epoxyeicosatrienoic acid (EET), 11,12‐EET, 14,15‐EET, 20‐hydroxyeicosatetraenoic acid (HETE), and inflammatory factors secreted by M1 macrophages including tumor necrosis factor alpha (TNF‐α), interleukin 1 beta (IL‐1β), inducible nitric oxide synthase (iNOS) were detected using ELISA kits (Cbybio, Nanjing, China) according to the kits' instructions.

### RT‐PCR detection and Western blot analysis

CYP2A6 mRNA expression was detected by RT‐PCR. Total RNA extraction was conducted using TRIzol (Thermo Fisher, Waltham, MA, USA), and the first‐strand cDNA was synthesized using a QuantiTect® Reverse Transcription Kit (Qiagen, Hilden, Germany). GAPDH was used as a control, and cDNA was cloned using PowerUp SYBR Green Master Mix Kit (Thermo Fisher) with specific primers (GAPDH: CCATGACAACTTTGGTATCGTGGAAGGCCATCACGCCACAGTTTC, CYP2A6: GCACAGTCTCCAATGTCATCAGCGCAACAGTGACAGGAACTCTT). CYP2A6 protein was detected using a western blot. Total protein was extracted from cells using RIPA lysis buffer (Beyotime, Shanghai, China). Western blotting was performed according to a standard method. The same amounts of total protein were isolated and incubated with primary antibodies CYP2A6 (Abcam, Cambridge, MA, USA). Western blot band intensities were analyzed using imagej software V1.6.0 (W.S. Rasband; NIH, Bethesda, MD, USA).

### Immunohistochemical staining of macrophage markers and CYP2A6

The immunohistochemical staining for CD206 and CD86 was performed using standard protocols. Briefly, liver tumor tissue sections were deparaffinized and antigen retrieved. Then, sections were incubated with antibodies against CD86 (Santa Cruz Biotechnology, Santa Cruz, CA), CD206 (Santa Cruz Biotechnology) overnight at 4 °C. After incubation, the sections were incubated with HRP‐conjugated secondary antibody (Beyotime) for 30 min at room temperature. Then, sections were developed with diaminobenzidine for 10 min and counterstained with hematoxylin for 1 min. Immunofluorescence staining of F4/80 was performed using the same methods with F4/80 antibody (Abcam). Nuclei were stained with 4',6‐diamidino‐2‐phenylindole (Beyotime) and observed by confocal laser scanning microscopy (LSM 880; Carl Zeiss, Oberkochen, Germany).

### Statistical analysis

Data were analyzed using spss 17.0 (SPSS Inc., Chicago, IL, USA). Comparisons between two groups were conducted using the unpaired Student's *t*‐test, and the Mann–Whitney *U*‐test. Comparisons among multiple groups were performed using one‐way analysis of variance (ANOVA) followed by Tukey's *post hoc* test. KM plot was conducted using r software (R Core Team, Vienna, Austria) and log‐rank test was used to calculate the *P*‐values. Cell composition comparisons between different groups were determined using a two‐sided Wilcoxon test. *P* < 0.05 was considered statistically significant.

## Results

### CYP2A6 expression in HCC

**Fig. 1:**
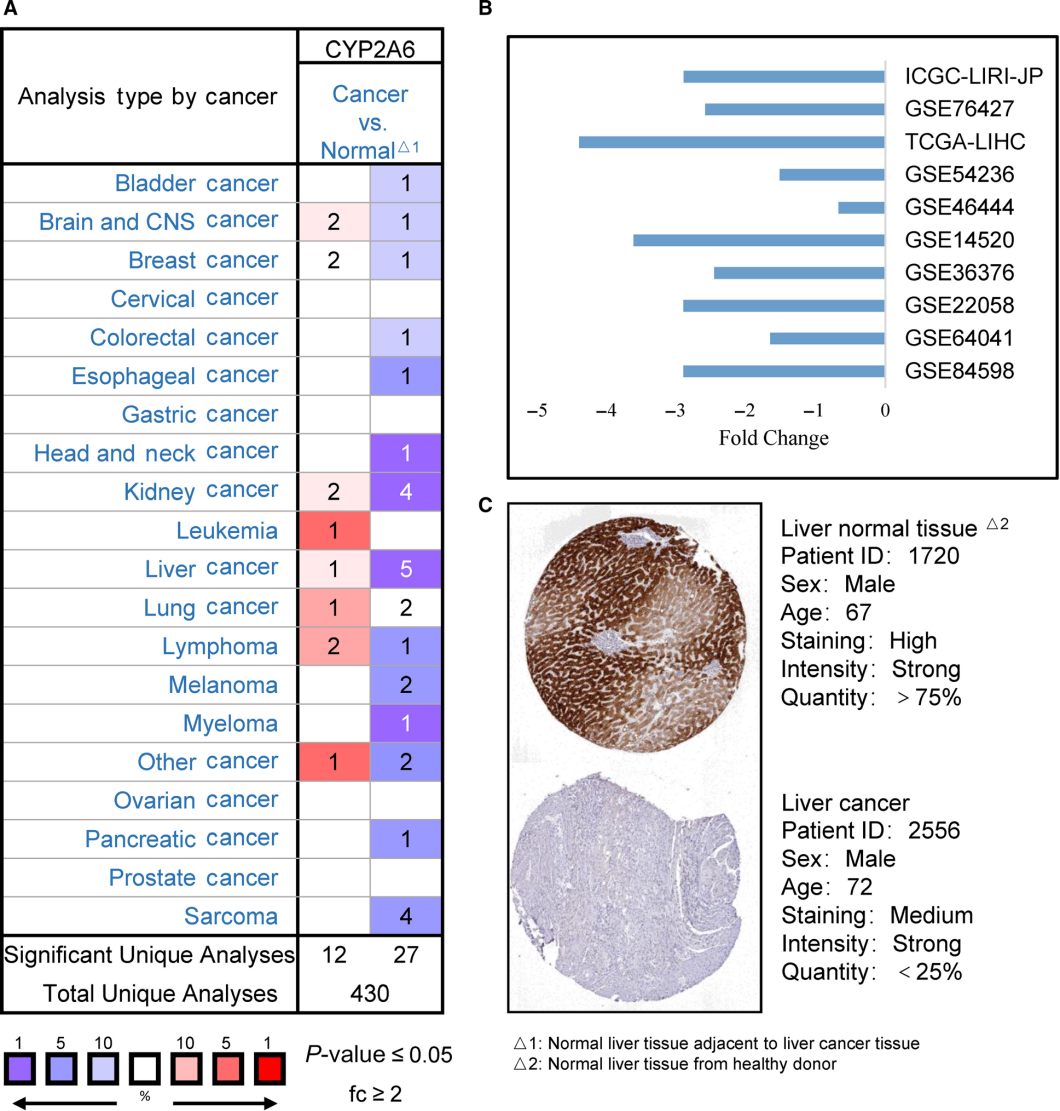
(A) Pan‐cancer analysis of CYP2A6 expression (Oncomine database). The graphic shows the number of datasets with statistical significance. Red cells indicate upregulation; violet cells indicate downregulation. The number in each cell represents the qualified datasets. The shade of the cell color indicates the highest gene rank percentile. (B) mRNA expression of CYP2A6 in multiple datasets. (C) Representative images of IHC staining of CYP2A6 from the HPA database.

The expression of CYP2A6 was significantly downregulated in most cancer types, especially in HCC patients (Fig. 1A). A similar pattern of expression was found in 10 different databases (Fig. 1B). Furthermore, there was a significant downregulation of CYP2A6 expression in different HCC histologies (Table 1). The immunostaining level of CYP2A6 in the HPA database showed a large decrease in CYP2A6 protein expression in HCC tissue sections compared to normal liver tissue from healthy donor (Fig. 1C). Overall, these data demonstrate that CYP2A6 is downregulated in HCC, suggesting it may play a protective role against HCC and may serve as a potential diagnostic indicator.

**Table 1:** The changes in CYP2A6 expression levels in normal liver tissues and different liver pathologies. FNH, focal nodular hyperplasia; ICC, intrahepatic cholangiocarcinoma; LCP, liver cancer precursor; normal, normal liver tissue samples adjacent to cancer.

	Tissue type	Fold change	*P* value	*t‐*test	Rank
CYP2A6	HCC vs Normal	−11.34	7.21E‐72	−23.86	85 (in top 1%)
	FNH vs Normal	−2.00	6.00E‐03	−3.68	108 (in top 1%)
	HCC vs Normal	−6.534	6.56E‐15	−8.62	348 (in top 4%)
	HCC vs Normal	−7.59	1.75E‐08	−7.61	276 (in top 3%)
	HCC vs Normal	−9.30	9.69E‐04	−3.56	1383 (in top 8%)
	HCC vs LCP	−3.70	2.24E‐06	−5.42	25 (in top 1%)
	HCC vs LCP	−10.93	3.83E‐09	−7.14	266 (in top 2%)
	Combined HCC and ICC vs HCC	−5.21	7.00E‐03	−3.06	791 (in top 7%)

### Associations between CYP2A6 expression and clinicopathological parameters in HCC

**Fig. 2:**
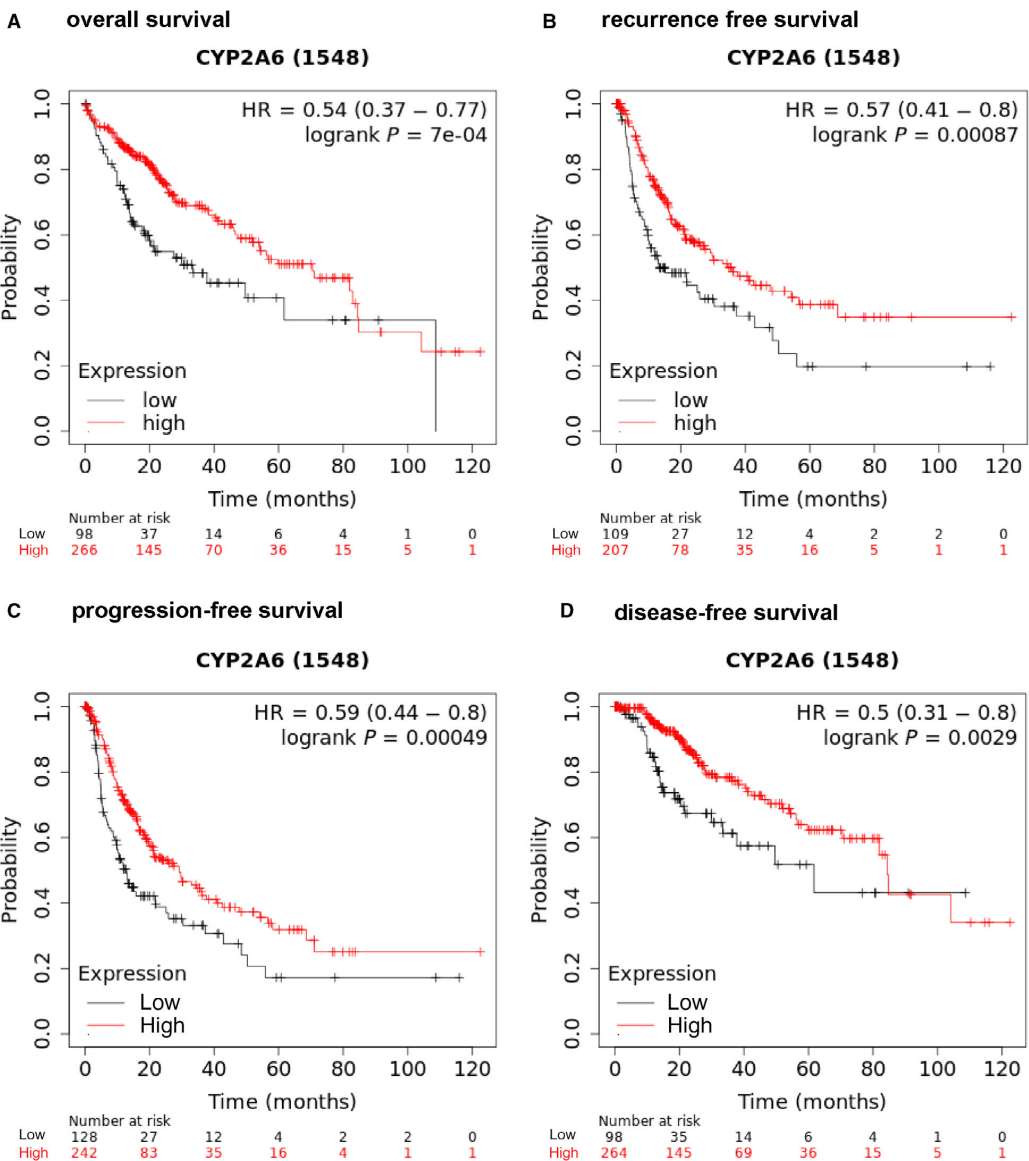
Relationship between survival time and expression of CYP2A6 in HCC patients. KM plots for (A) OS, (B) RFS, (C) PFS, and (D) DFS.

The group with lower levels of CYP2A6 expression had significantly shorter overall survival (OS), relapse‐free survival (RFS), progression‐free survival (PFS), and disease‐free survival (DFS) (Fig. 2A–D). Several clinicopathological parameters, including histologic grade, pathologic stage, pathologic T, and pathologic N, were related to the expression of CYP2A6. Lower expression of CYP2A6 was associated with a more serious pathological grade, and there was a negative correlation between CYP2A6 expression and vascular metastasis. Furthermore, patients with hepatitis, liver inflammation, and low body mass index also exhibited lower CYP2A6 expression levels (Table 2). These data strongly suggest that CYP2A6 is closely related to the progression of HCC and is a key gene affecting its progression.

**Table 2:** Association between CYP2A6 expression and clinicopathological parameters in HCC.

Clinicopathological	*N*	CYP2A6 expression Mean ± SD	*P* value
Gender			
Male	250	20.26 ± 4.285	0.2294
Female	121	19.63 ± 4.532	
Histologic grade			
G1	55	21.94 ± 3.376	< 0.0001***
G2	177	20.78 ± 4.250	
G3	122	18.39 ± 4.198	
G4	12	17.11 ± 4.665	
Pathologic stage			
S1	171	21.02 ± 3.796	< 0.0001***
S2	86	19.33 ± 4.323	
S3	85	18.42 ± 4.914	
S4	5	18.43 ± 5.325	
Pathologic T			
T1	181	21.06 ± 3.769	< 0.0001***
T2	94	19.18 ± 4.522	
T3	80	18.39 ± 4.928	
T4	13	21.08 ± 3.592	
Pathologic N			
No	252	19.59 ± 4.428	0.0234
Yes	4	14.52 ± 2.679	
Pathologic M			
No	266	19.54 ± 4.448	0.8587
Yes	4	19.14 ± 5.869	
Hepatitis			
No	127	20.30 ± 4.358	0.0372*
Yes	164	19.48 ± 4.734	
Fibrosis score			
0	74	20.99 ± 4.440	0.7128
1–2	31	20.39 ± 3.924	
3–4	28	20.00 ± 4.255	
5–6	79	20.59 ± 3.672	
BMI			
< 18	12	16.59 ± 0.9112	< 0.0001***
18–24	146	21.56 ± 1.659	
24–28	84	25.86 ± 1.122	
28	93	33.58 ± 5.549	
Vascular metastasis			
None	206	20.85 ± 3.964	< 0.0001***
Micro	93	19.40 ± 4.129	
Macro	16	16.93 ± 4.720	
Liver inflammation			
None	117	21.46 ± 3.827	
Mild	117	19.66 ± 4.147	0.0029**
Severe	18	20.14 ± 4.135	

Asterisks represent significant differences between groups (*P < 0.05, **P < 0.01, ***P < 0.001, ANOVA.)

### Analysis of CYP2A6 and immune cell infiltration

**Fig. 3:**
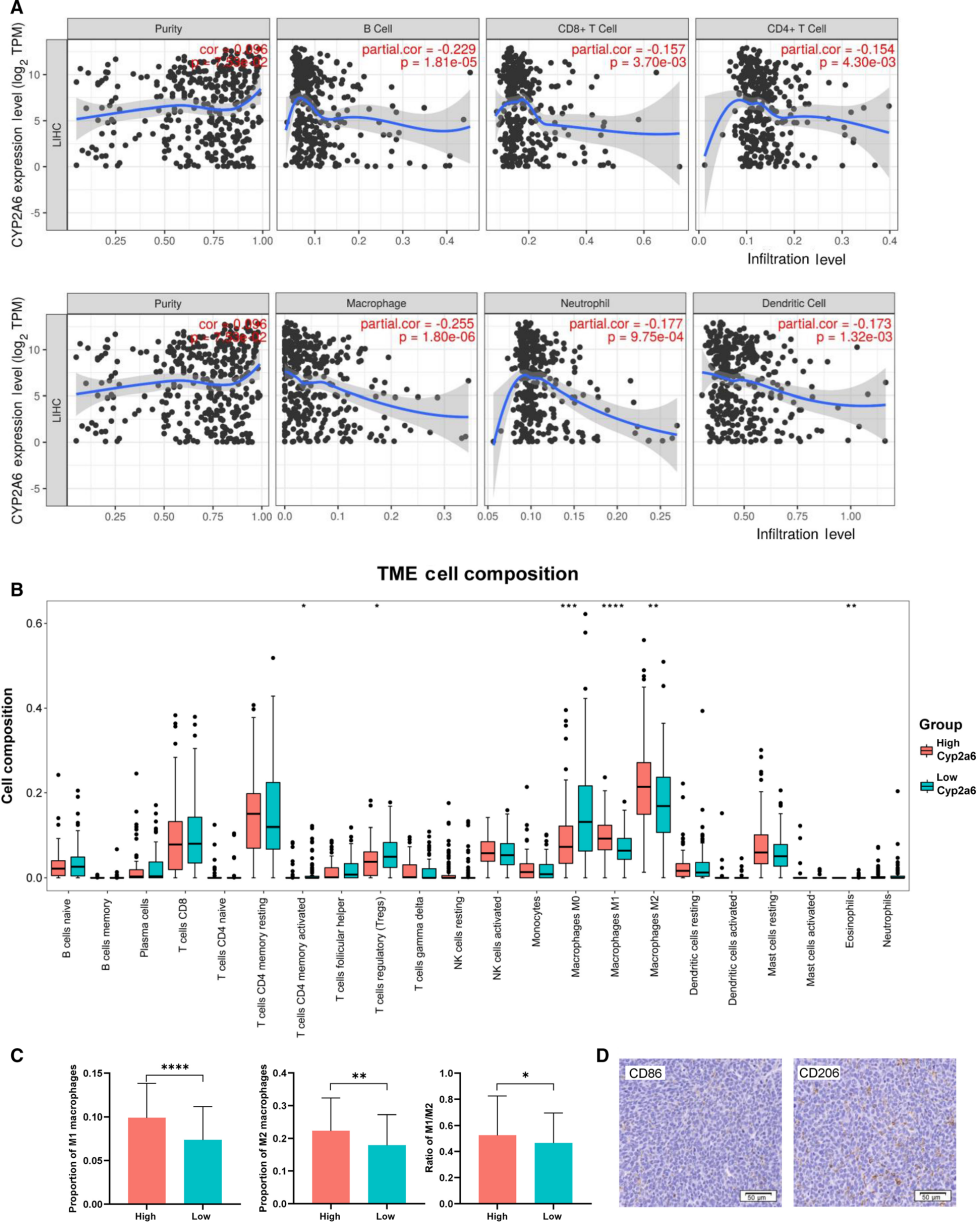
Immune cell infiltration associated with CYP2A6. (A) Relationship between infiltration of six major immune cells and expression of CYP2A6 calculated by TIMER. (B) Overview of absolute infiltration scores of 22 immune cell types in the high and low CYP2A6 expression groups calculated by CIBERSORT. Macrophages had the highest proportion of infiltration. In addition, in the low CYP2A6 expression group, the proportion of nonactivated macrophages (M0) was significantly increased, and activated macrophages (M1 and M2) were significantly decreased. *P < 0.05, **P < 0.01, ***P < 0.001, ****P < 0.0001, Wilcoxon test. Mean ± SD for *n* = 93. (C) Proportion of different types of macrophages in the high and low CYP2A6 expression groups according to CIBERSORT. Elevation of CYP2A6 was associated with macrophage activation (elevated ratio of M1 and M2 infiltration) and was proportionally more prone to M1‐type macrophages (elevated ratio of M1/M2). *P < 0.05, **P < 0.01, ***P < 0.001, ****P < 0.0001, Student's *t*‐test. Mean ± SD for *n* = 89. (D) Immunohistochemical staining of M1 marker CD86 and M2 marker CD206 in tumor tissues of mice. Scale bar = 50 μm.

We analyzed the immune cell infiltration using two independent databases. Timer database results showed that immune cells related to CYP2A6 were macrophages, B cells, and neutrophils (Fig. 3A). The results calculated by CIBERSORT algorithm indicated that the proportions of regulatory T cells, activated CD4 memory T cells, eosinophils, and macrophages change depending on the expression level of CYP2A6. In particular, the change in macrophage phenotype showed the greatest significant difference with *P* < 0.0001 (Fig. 3B). In the group with high CYP2A6 expression, the proportion of nonactivated macrophages (M0) was lower, and the amounts of M1 and M2 macrophages were higher. Furthermore, the ratio of M1/M2 was elevated in the high CYP2A6 expression group (Fig. 3C). To further verify the infiltration of different macrophages in tumor cells, we examined the expression of CD86 (marker for M1 macrophage) and CD206 (marker for M2 macrophage) in tumor tissues. The results showed that the positive rate of CD206 was significantly higher than CD86 (Fig. 3D). These data suggested a critical role for macrophages in tumor progression and CYP2A6 expression is positively correlated with macrophage activation and may contribute to the transformation of M1 phenotype.

### TSIIA activates CYP2A6 and regulates macrophage polarization

**Fig. 4:**
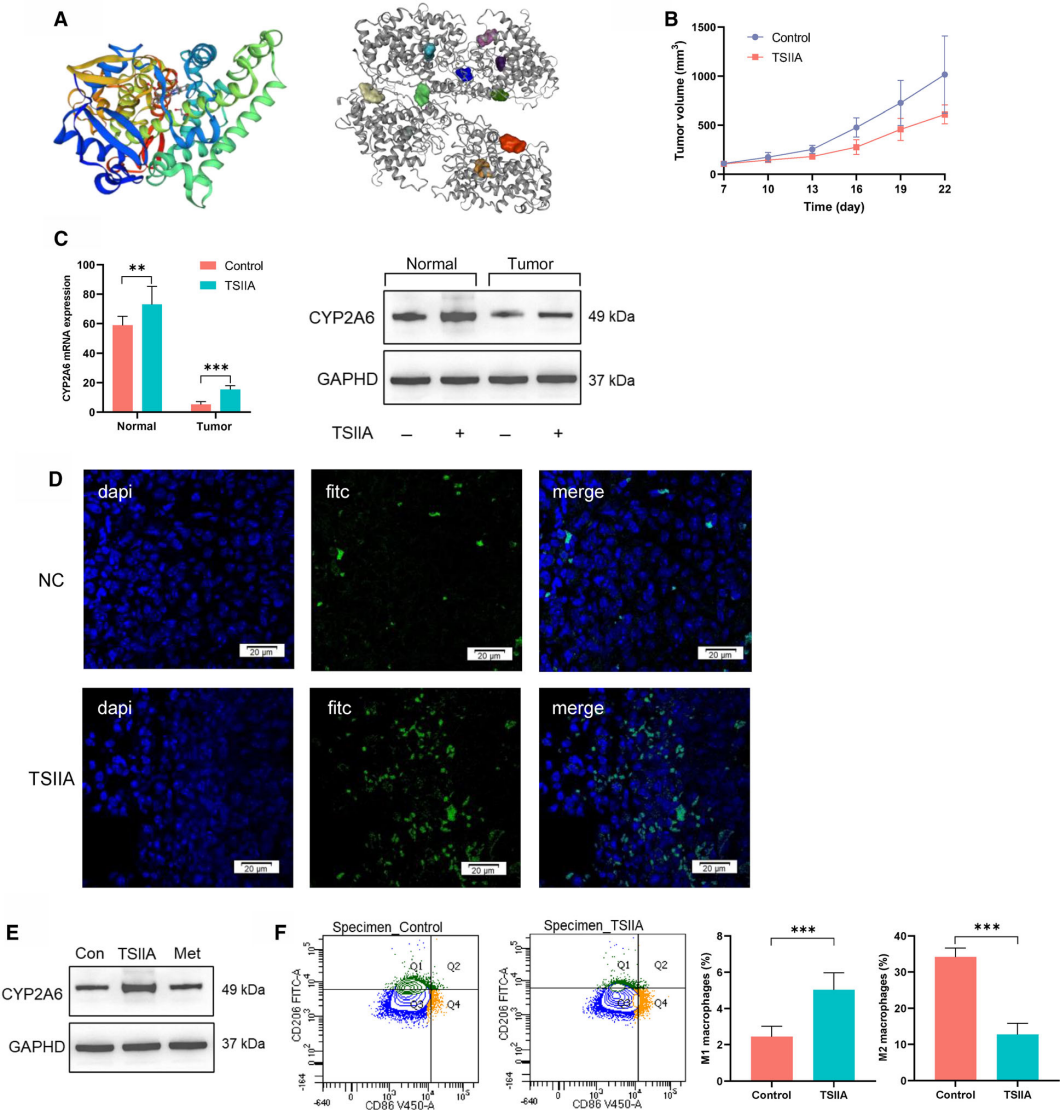
TSIIA can regulate CYP2A6 and affect macrophage polarization. (A) The structure of CYP2A6 protein (left) and the docking between TSIIA and CYP2A6 (right). Top 10 binding sites are shown. (B) Growth curve of tumor volume measured on different days. (C) Expression of CYP2A6 mRNA and protein in normal liver tissue and tumor tissue. **P < 0.01, ***P < 0.001, Student's *t*‐test. Mean ± SD for *n* = 3. (D) Immunofluorescence staining identified that TSIIA could increase the expression of CYP2A6 in tumor tissues. CYP2A6 was labeled with FITC. (E) Expression of CYP2A6 protein in Hep1‐6 cells with different treatment. Scale bar = 20 μm. (F) Changes in the proportions of M1 and M2 macrophages in the control and TSIIA groups. Cells in Q1 represent the M2 macrophages and cells in Q4 represent the M1 macrophages. Cells in Q3 represent the M0 macrophages. ***P < 0.001, Student's *t*‐test. Mean ± SD for *n* = 3.

The above analysis indicates that CYP2A6 acts as a protective factor for HCC and leads to changes in macrophage polarization, but no effective activators of CYP2A6 have been identified so far. We screened the extracts of *S. miltiorrhiza* using the TCMSP database and obtained the predictive targets for each compound. The results showed that TSIIA has the most possibility to be a regulator of CYP450 family (Table S1). Molecular docking studies disclosed that TSIIA has a good binding affinity for CYP2A6 (Fig. 4A). To verify this result, we established a transplantation tumor model of liver cancer in mice and used TSIIA for 21 days of intervention treatment. The results showed that TSIIA had an inhibitory effect on liver tumor growth (Fig. 4B). We detected the expression of CYP2A6 in tumor tissues and normal liver tissues, with the expression of CYP2A6 in normal liver tissues significantly enhanced compared to tumor tissues, consistent with the IHC staining data from the HPA database. Not only that, TSIIA could increase CYP2A6 mRNA and protein levels in both normal liver tissue and tumor tissue (Fig. 4C). Immunofluorescence of tumor tissues also showed that TSIIA could improve the infiltration and expression of CYP2A6 in tumor tissues (Fig. 4D). Then, we treated hepatoma cells with TSIIA to verify the regulation of CYP2A6, and the results showed that TSIIA increased the expression of CYP2A6 in Hep1‐6 cells cultured *in vitro* (Fig. 4E). This revealed that TSIIA was the positive activator of CYP2A6 and can play an antitumor role by increasing the expression of CYP2A6. We further examined the polarization of macrophages; consistent with the results in Fig. 3, typing analysis of tumor‐associated macrophages (TAMs) revealed that the phenotype in the HCC tumor tissue was mainly M2, whereas, in the TSIIA group, the proportion of M1 macrophage phenotype was increased (Fig. 4F). All these data suggested that TSIIA can promote CYP2A6 expression and M1 macrophage polarization, and there seems to be a close relationship between them.

### CYP2A6 affects macrophage polarization through AA metabolism

**Fig. 5:**
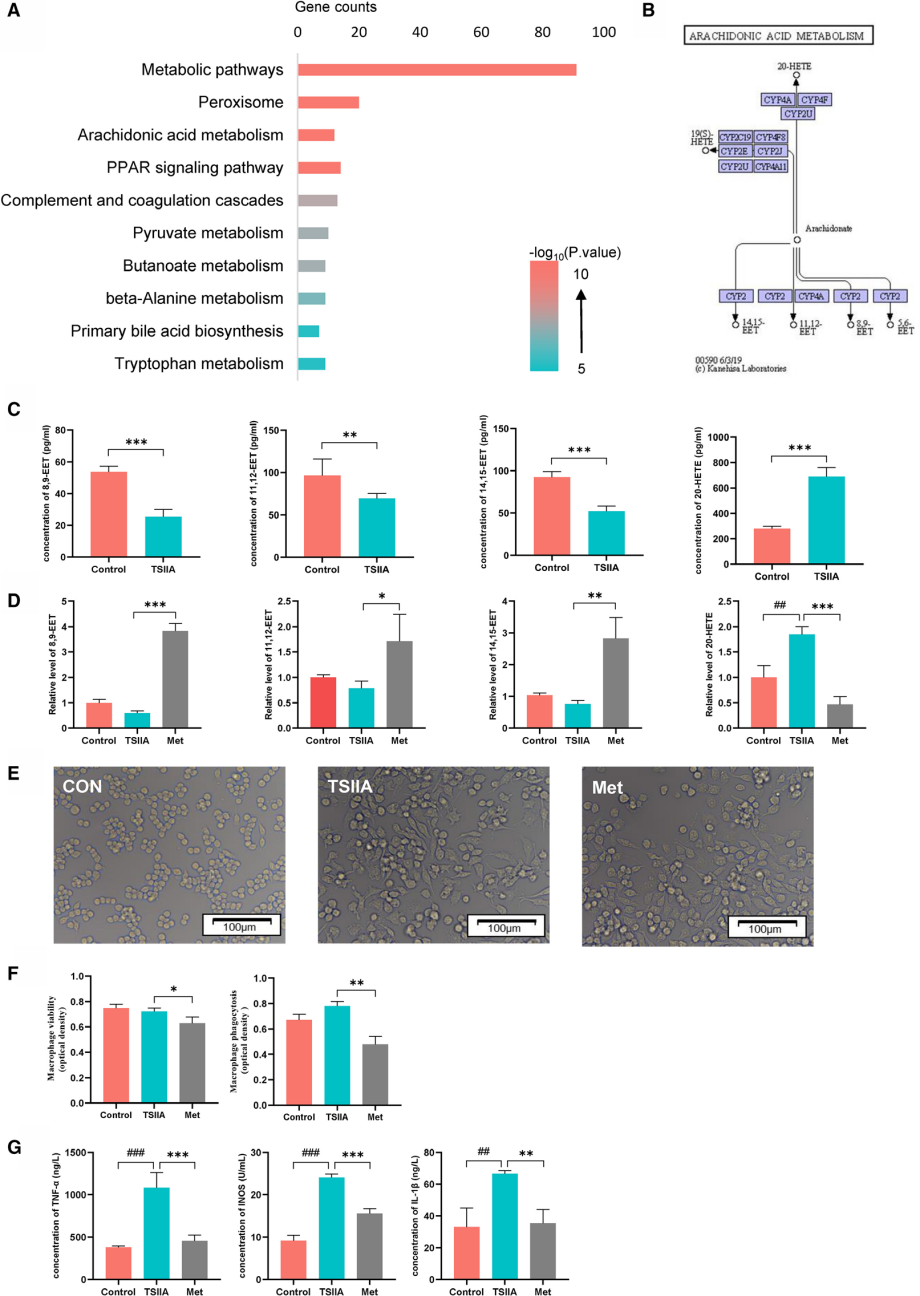
(A) Prediction of KEGG pathway related to CYP2A6. (B) AA can be metabolized into EETs and HETEs by different CYP450 members (data were collected from the KEGG database and the image was slightly modified for better visualization). (C) AA metabolites in control group and TSIIA group *in vivo*. **P < 0.01, ***P < 0.001, Student's *t*‐test. Mean ± SD for *n* = 6. (D) AA metabolites in cell supernatant of control group, TSIIA group, and Met group. *^##^P* < 0.01 vs control; *P < 0.05, **P < 0.01, ***P < 0.001 vs TSIIA; ANOVA. Mean ± SD for *n* = 6. (E) Macrophages under different drug interventions. Inactivated macrophages are oval. Activated macrophages show numerous pseudopods emanating from their surfaces and become spindled in shape. Scale bar = 100 μm. (F) Viability (left) and phagocytosis of macrophages (right) in different groups. *P < 0.05, **P < 0.01, ANOVA. Mean ± SD for *n* = 3. (G) Analysis of representative proinflammatory factors of M1 macrophage. ^##^ *P* < 0.01, ^###^ *P* < 0.001, vs control; **P < 0.01, ***P < 0.001 vs TSIIA. Mean ± SD for *n* = 3.

To understand the relationship between CYP2A6 and macrophage polarization, we first analyzed the genes closely related to CYP2A6 using bioinformatics methods and try to find the enriched functions they participated in. The results showed that CYP2A6 was closely related to peroxisome and AA metabolism (Fig. 5A). Previous studies have shown that the CYP450 family can metabolize AA into different products (Fig. 5B) and that they can lead to changes in macrophage polarization [16, 17]. Therefore, we first detected the metabolites of AA in the serum of mice *in vivo*. AA metabolite assays showed that TSIIA treatment resulted in an increase in 20‐HETE and a decrease in 8,9‐EET, 11,12‐EET, and 14,15‐EET (Fig. 5C). To verify whether this phenomenon was caused by the elevation of CYP2A6, we retested it *in vitro* and used inhibitors of CYP2A6 as a control. As with the *in vivo* results, TSIIA interference resulted in an increase in 20‐HETE and a decrease in 8,9‐EET, 11,12‐EET, and 14,15‐EET, and these changes can be blocked by CYP2A6 inhibitors (Fig. 5D). This suggested that CYP2A6 was responsible for AA metabolism which mainly metabolizes AA to form 20‐HETE. To further investigate the effects of these metabolites on macrophages, we incubated macrophages using conditioned medium with different AA metabolites. After 48 h of culture, the microscopic photography showed that different AA metabolites could cause changes in macrophage phenotype with numerous pseudopods emanating from their surfaces (Fig. 5E). We examined the activity of macrophages with CCK8 and found that inhibition of CYP2A6 resulted in a decrease in macrophage viability (Fig. 5F). At the same time, reducing of EETs and increasing of 20‐HETE improving macrophage phagocytosis, and this can be blocked by CYP2A6 inhibitors (Fig. 5F). We further examined the expression of M1 characteristic secretory factors in the supernatant. The results showed that the inflammatory markers of M1 including TNF‐α, IL‐1β, INOS were elevated after TSIIA treatment, while methoxsalen inhibited the elevation (Fig. 5G). This strongly suggested that CYP2A6 regulated by TSIIA promotes M1 macrophage activation by regulating AA metabolism.

## Discussion

**Fig. 6:**
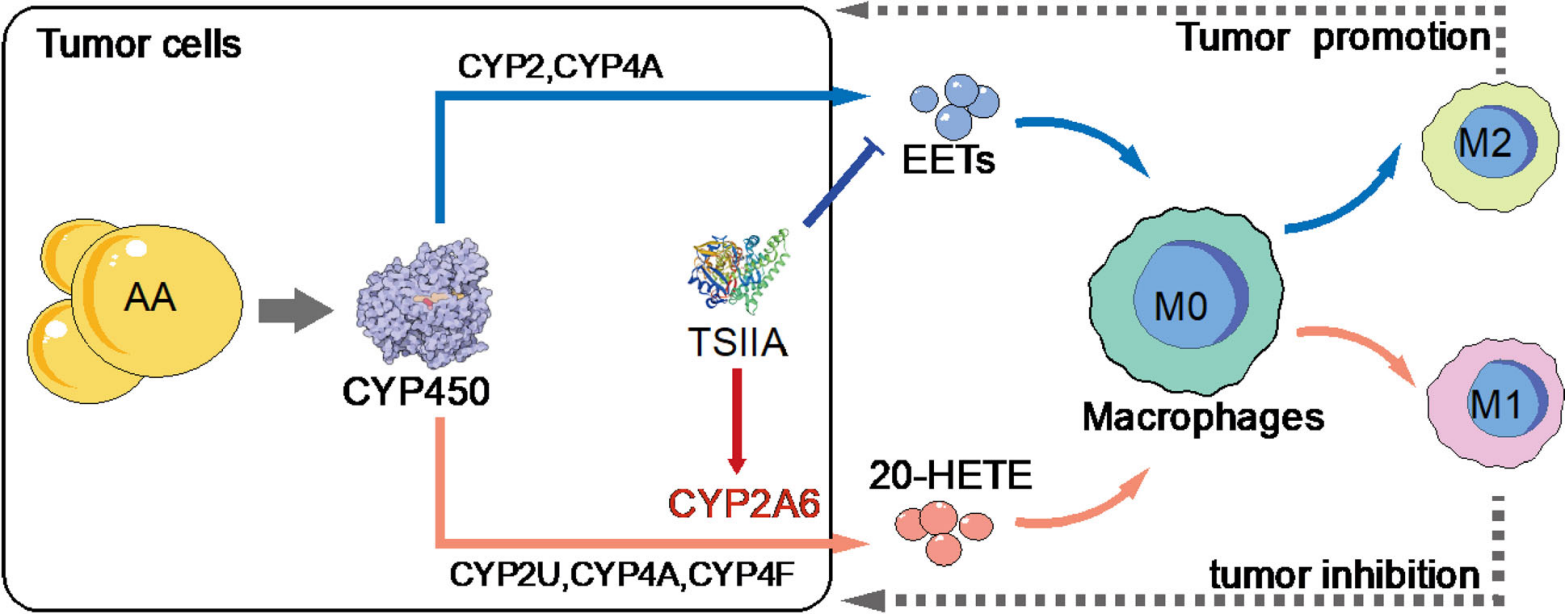
TSIIA plays an antitumor role by regulating the polarization of macrophages through CYP2A6. AA can be metabolized by different CYP450 family members and transformed to different metabolites. The blue line shows one of the paths: several CYP2 members and CYP4A metabolize AA to generate EETs. EETs promote M2 polarization of macrophages and promote tumor progression. The red line shows another line in which the CYP450 family is involved. CYP2A6 and other CYP450 members metabolize AA to 20‐HETE, which further induces the polarization of M1 macrophages. M1 can play an antitumor role in a variety of ways. TSIIA can increase the expression of CYP2A6, thus promoting the generation of 20‐HETE and inhibiting EETs to exhibit an antitumor effect.

In this study, we explored the expression of CYP2A6 in HCC and evaluated the correlation between CYP2A6 and immunity. Our results indicate that the expression level of CYP2A6 is reduced in HCC, which affects the polarization of macrophages, and creates a tumor microenvironment conducive to tumorigenesis. Mechanistically, CYP2A6 participates in the metabolism of AA, reduces the production of EETs, and increases the production of 20‐HETE. Dysregulation in AA metabolites can lead to changes in macrophage activity and phagocytosis and affect macrophage polarization. TSIIA increases the activation and polarization of macrophages through CYP2A6, which may help to achieve the goal of treating liver cancer. A brief overview of overall mechanism is shown in Fig. 6.

CYP2A6 is an important member of the CYP450 metabolic family and is differentially expressed in different tumors. In colorectal tumors [18] and lung cancers [19], the expression of CYP2A6 is upregulated and associated with the degree of malignancy of the tumor. However, some studies have shown opposing results. In this study, we identified CYP2A6 as a protective factor for HCC patients.

The expression of CYP2A6 has obvious genetic polymorphism and is induced by a variety of substances including drugs, endogenous substances, and dietary components [20]. Therefore, it is necessary to identify whether CYP2A6 fluctuations mainly originate from HCC progression. In the analysis of clinical parameters, we found a close relationship between CYP2A6 and pathological grading grades of HCC, and the expression of CYP2A6 markedly decreased with the advanced grading. Since CYP2A6 is mainly expressed in hepatocytes, the decrease in normal hepatocytes caused by HCC may cause the reduction of CYP2A6. However, the subsequent analysis showed that the expression of CYP2A6 was not correlated with the degree of liver fibrosis and only showed a slight correlation with patients with hepatitis, indicating that the simple hepatocellular injury and decrease in normal hepatocytes was not the main reason for CYP2A6 reduction. In particular, the expression of CYP2A6 decreased dramatically in patients with macrovascular metastases and severe liver inflammation, suggesting that CYP2A6 is mainly affected by the progression of liver cancer and is involved in the inflammatory microenvironment of HCC. The close relationship between CYP2A6 and clinical parameters makes it have the potential to become a new biomarker for HCC, and it is also one of the effective therapeutic targets for liver cancer patients.

In addition to the close association between CYP2A6 and HCC, more important, CYP2A6 was closely related to macrophage polarization which is rarely reported in the literature. This interconnects metabolism with immunity and offers possibilities in the treatment of HCC. TAMs are the most infiltrating cells in the tumor microenvironment and are closely associated with the invasion and metastasis of tumors. Macrophages are generally classified into two types: classically activated (M1) and alternatively activated (M2). M1 macrophages exert phagocytic and antitumor effects, while M2 macrophages promote tumor progression [21]. In our study, we found that high expression of CYP2A6 promoted macrophage activation and was positively correlated with M1 polarization. The normal expression of CYP2A6 is necessary for the viability and phagocytosis of macrophages. This provides a reasonable explanation for CYP2A6 intervention in tumor progression. Since CYP2A6 is an important metabolic gene, we speculate that these changes are closely related to the metabolic reprogramming caused by CYP2A6. As a super metabolism family, AA metabolism is one of the major functions of the CYP450 family [22] and is closely related to tumor progression. Specifically, AA can be metabolized by different CYP2A family members into two types of metabolites, namely HETE and EETs. Our study suggested that CYP2A6 was involved in the generation of 20‐HETE rather than EETs. The aberrant expression of CYP2A6 can shift the equilibrium of 20‐HETE and EETs, leading to an increase in 20‐HETE and a decrease in EETs. Both HETE and EETs were found to play a central role in the progression of tumor [23]. In addition, some studies have shown a direct relationship between AA metabolites and macrophages. EETs have proven to be one of the stimulators of M2 macrophages, which can inhibit the proinflammation gene production by activating peroxisome proliferator‐activated receptors and inhibiting the nuclear factor kappa B signaling pathway [24]. 20‐HETE is an important signaling eicosanoid involved in the regulation of angiotensin and angiogenesis. 20‐HETE can activate key protein kinases, proinflammatory mediators, chemokines in cancer, and play a proinflammatory role in tumor progression [25, 26]. However, the role of 20‐HETE to macrophages is little known. Our study showed that elevation of 20‐HETE promotes M1 transformation, promotes the production of proinflammatory cytokines, and enhances macrophages phagocytosis. Macrophage phagocytosis is important for tumor killing and antigen presentation. This is a positive result in the aim of antitumor. M1 macrophages and their secretion of proinflammatory cytokines are important for immune regulation. Thus, TAM‐targeted therapies are intensively being studied and one of the therapeutic strategies is to reprogram TAM to M1 macrophages. Our study provides a useful complementary approach to targeting TAM against tumors, emphasizing that macrophages can be modulated by cell metabolism, thereby affecting the immune environment.

The balance of AA metabolites regulated by CYP2A6 is important for maintaining normal immunity.

Due to the important role of CYP2A6, we sought to find drugs that could interfere with CYP2A6. At present, dexamethasone and rifampicin have been shown to induce CYP2A6 activity and expression by increasing the transcription of CYP2A6 [27, 28]. However, there is no robust research evidence to support their application in tumor. TSIIA is a typical drug used in traditional Chinese medicine to regulate metabolism and has also been explored in the treatment of HCC [29, 30]. Extensive research has been conducted on TSIIA and makes TSIIA clinical application possible. Here, we showed that TSIIA increased the expression of CYP2A6 therefore affects AA metabolism and macrophage polarization. This provides some insight into the molecular mechanisms of the antitumor effect of TSIIA.

In conclusion, we have identified CYP2A6 as a protective factor in HCC, which may also serve as a new biomarker of HCC. As a member of the metabolic family, CYP2A6 can affect the immune environment of the body, especially by the regulation of macrophages. TSIIA can regulate the expression of CYP2A6, thereby increasing macrophage polarization. Our study links metabolism and immunity, providing evidence for its complex relationship. The ability to develop drugs by studying the relationship between metabolism and immunity is a promising research direction.

## Conflict of interest

The authors declare no conflict of interest.

## Author contributions

GZ and ZL conceived and designed the project. TJ performed experiments and wrote the manuscript. AZ provided technical support and performed data analysis. CY, CX, and DY contributed to the data collection. All authors read and approved the final manuscript.

## Supporting information


**Table S1.** Predicted targets of molecules obtained from *Salvia miltiorrhiza*.

## Data Availability

The data will be available from the corresponding author upon reasonable request.
